# [Corrigendum] The osteoarthritis‑associated gene PAPSS2 promotes differentiation and matrix formation in ATDC5 chondrogenic cells

**DOI:** 10.3892/etm.2024.12684

**Published:** 2024-08-08

**Authors:** Liying Fan, Yuan He, Jing Han, Puwei Yuan, Xiong Guo, Weizhuo Wang

Exp Ther Med 16:5190–5200, 2018; DOI: 10.3892/etm.2018.6843

Subsequently to the publication of this paper, an interested reader drew to the authors’ attention that, for the immunocytochemical staining experiments shown in Figs. 1 and 3 on p. 5194 and p. 5195 respectively, the ‘Control’ data panel in Fig. 1C and the ‘Col X / PS’ panel in Fig. 3G were partially overlapping, such that data which were intended to show the results from differently performed experiments had apparently been derived from the same original source.

After having re-examined their original data, the authors realized that the data panel shown in Fig. 1C had inadvertently been selected incorrectly. A revised version of Fig. 1, now featuring the correct data for the ‘Col X / PS’ panel in Fig. 3G, is shown below. Note that this error did not have a major impact on the conclusions reported in this study, and all the authors agree to the publication of this Corrigendum. The authors thank the editor of *Experimental and Therapeutic Medicine* for granting them the opportunity to publish this Corrigendum, and apologize to the readership for any inconvenience caused.

## Figures and Tables

**Figure 3:**
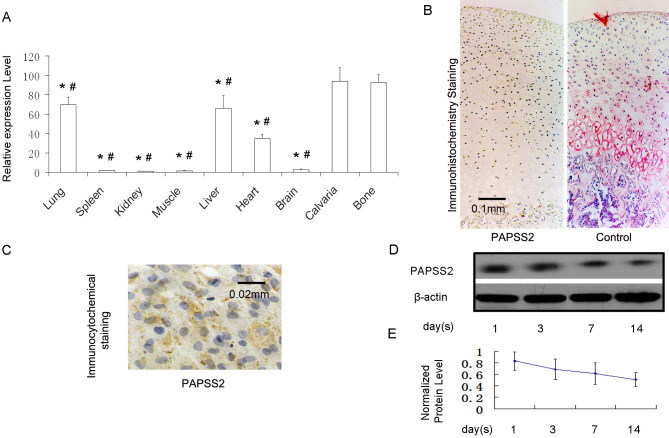
Effect of PAPSS2 expression on extracellular matrix formation. Upon reaching 80% subconfluency, ACTD5 cells were grown in differentiation media containing either lentiviral plasmid expressing PAPSS2 small hairpin RNA for knockdown or pBMN-PAPSS2 vector for overexpression of PAPSS2 for 14 days. (A-C) Alcian blue staining and immunocytochemical staining for (D-F) Col types II and (G-I) X in the PO, PS and control chondrocytes was performed, respectively (magnification, ×400). Col, collagen; PO, PAPSS overexpression group; PS, PAPSS suppression group; PAPSS2, 3’-phosphoadenosine 5’-phosphosulfate synthetase 2.

